# Shared B cell memory to coronaviruses and other pathogens varies in human age groups and tissues

**DOI:** 10.1126/science.abf6648

**Published:** 2021-04-12

**Authors:** Fan Yang, Sandra C. A. Nielsen, Ramona A. Hoh, Katharina Rltgen, Oliver Fabian Wirz, Emily Haraguchi, Grace H. Jean, Ji-Yeun Lee, Tho D. Pham, Katherine J. L. Jackson, Krishna M. Roskin, Yi Liu, Khoa Nguyen, Robert S. Ohgami, Eleanor M. Osborne, Kari C. Nadeau, Claus U. Niemann, Julie Parsonnet, Scott D. Boyd

**Affiliations:** 1 Department of Pathology, Stanford University, Stanford, CA 94305, USA.; 2 Stanford Blood Center, Stanford University, Stanford, CA 94305, USA.; 3 Garvan Institute of Medical Research, Darlinghurst, NSW 2010, Australia.; 4 Department of Pediatrics, University of Cincinnati, Cincinnati, OH 45267, USA.; 5 Division of Biomedical Informatics, Cincinnati Childrens Hospital Medical Center, Cincinnati, OH 45229, USA.; 6 Division of Immunobiology, Cincinnati Childrens Hospital Medical Center, Cincinnati, OH 45229, USA.; 7 Calico Life Sciences, South San Francisco, CA 94080, USA.; 8 Department of Pathology, University of California, San Francisco, CA 94143, USA.; 9 Sarah Cannon Cancer Center, Tennessee Oncology, Smyrna, TN 37167, USA.; 10 Sean N. Parker Center for Allergy and Asthma Research, Stanford University, Stanford, CA 94305, USA.; 11 Division of Pulmonary, Allergy and Critical Care Medicine, Stanford University, Stanford, CA 94305, USA.; 12 Department of Anesthesia and Perioperative Care, University of California, San Francisco, CA 94143, USA.; 13 Department of Surgery, Division of Transplantation, University of California, San Francisco, CA 94143, USA.; 14 Department of Medicine, Stanford University, Stanford, CA 94305, USA.; 15 Epidemiology and Population Health, Stanford University, Stanford, CA 94305, USA.

## Abstract

Vaccination and infection promote the formation, tissue distribution, and clonal evolution of B cells, which encode humoral immune memory. We evaluated pediatric and adult blood and deceased adult organ donor tissues to determine convergent antigen-specific antibody genes of similar sequences shared between individuals. B cell memory varied for different pathogens. Polysaccharide antigenspecific clones were not exclusive to the spleen. Adults had higher clone frequencies and greater class switching in lymphoid tissues than blood, while pediatric blood had abundant class-switched convergent clones. Consistent with reported serology, prepandemic children had class-switched convergent clones to severe acute respiratory syndrome coronavirus 2 with weak cross-reactivity to other coronaviruses, while adult blood or tissues showed few such clones. These results highlight the prominence of early childhood B cell clonal expansions and cross-reactivity for future responses to novel pathogens.

## Kids armed with anti-coronavirus B cells

It remains unclear whether B cell repertoires against coronaviruses and other pathogens differ between adults and children and how important these distinctions are. Yang *et al.* analyzed blood samples from young children and adults, as well as tissues from deceased organ donors, characterizing the B cell receptor (BCR) repertoires specific to six common pathogens and two viruses that they had not seen before: Ebola virus and severe acute respiratory syndrome coronavirus 2 (SARS-CoV-2). Children had higher frequencies of B cells with convergent BCR heavy chains against previously encountered pathogens and higher frequencies of class-switched convergent B cell clones against SARS-CoV-2 and related coronaviruses. These findings suggest that encounters with coronaviruses in early life may produce cross-reactive memory B cell populations that contribute to divergent COVID-19 susceptibilities.

*Science*, this issue p. 738

## Teaser

Blood taken from children before the COVID-19 pandemic contains memory B cells that bind SARS-CoV-2.

The clonal identity of a B cell can be traced by the sequence of its B cell receptor (BCR), which determines its antigen specificity (*1*). Immunoglobulin (Ig) sequences are formed via irreversible variable, diversity, and joining (VDJ) gene segment rearrangement and can be diversified through somatic hypermutation (SHM) and class-switch recombination (CSR) (*2*). Convergent BCRs with high sequence similarity in individuals exposed to the same antigen reflect antigen-driven clonal selection and form shared immunological memory between individuals (*3*,*5*). It is still unclear, however, how B cell memory to different antigens distributes in human tissues and changes during an individuals life span.

Humoral immune responses can differ between children and adults; for example, children use more B cell clones to achieve neutralizing antibody breadth to HIV-1 (*6*). Children usually have milder disease following severe acute respiratory syndrome coronavirus 2 (SARS-CoV-2) infection than adults do (*7*,*10*), potentially owing to differences in viral receptor expression and immune responses (*11*, *12*). SARS-CoV-2infected children, in contrast to adults, show lower antibody titers and more IgG specific for the spike (S) protein over the nucleocapsid (N) protein. The faster viral clearance and lower viral antigen loads in children have been attributed to these differences (*13*). Whether B cell clones specific for coronaviruses and other pathogens differ between children and adults is unclear. Blood-based studies survey only a fraction of an individuals BCR repertoire. The lymph nodes, spleen, and gastrointestinal tract harbor greater numbers of B cells and are major sites for SHM and CSR (*14*, *15*). Specialized responses in particular tissues have been reported, such as for polysaccharide antigenspecific B cells in functional splenic tissue (*16*, *17*).

To study changes in antigen-specific B cell memory over the human life span and across tissues, we characterized convergent Ig heavy chain (IGH) repertoires specific to six common pathogens as well as two viruses not encountered by the participants, Ebola virus (EBOV) and SARS-CoV-2, in pre-COVID-19 pandemic individuals. We analyzed 12 cord blood (CB) samples; 93 blood samples from 51 children aged 1 to 3 years (*18*); 114 blood samples from healthy human adults aged 17 to 87 years (*18*); and blood, lymph node, and spleen samples from eight deceased organ donors (table S1). Children were vaccinated against *Haemophilus influenzae* type b (Hib), *Pneumococcus pneumoniae* (PP), and tetanus toxoid (TT) at 2, 4, 6, and 12 to 15 months, had influenza virus (flu) vaccination, and were very likely exposed to respiratory syncytial virus (RSV) but were not vaccinated against *Neisseria meningitidis* (NM) (*19*). Adult vaccination histories were unknown. Convergent IGHs were identified by clustering with pathogen-specific reference IGH (table S2) sharing IGH variable domain (IGHV) and joining region (IGHJ) gene segment usage, complementarity-determining region H3 (CDR-H3) length, and minimum 85% CDR-H3 amino acid sequence identity.

**Fig. 1:**
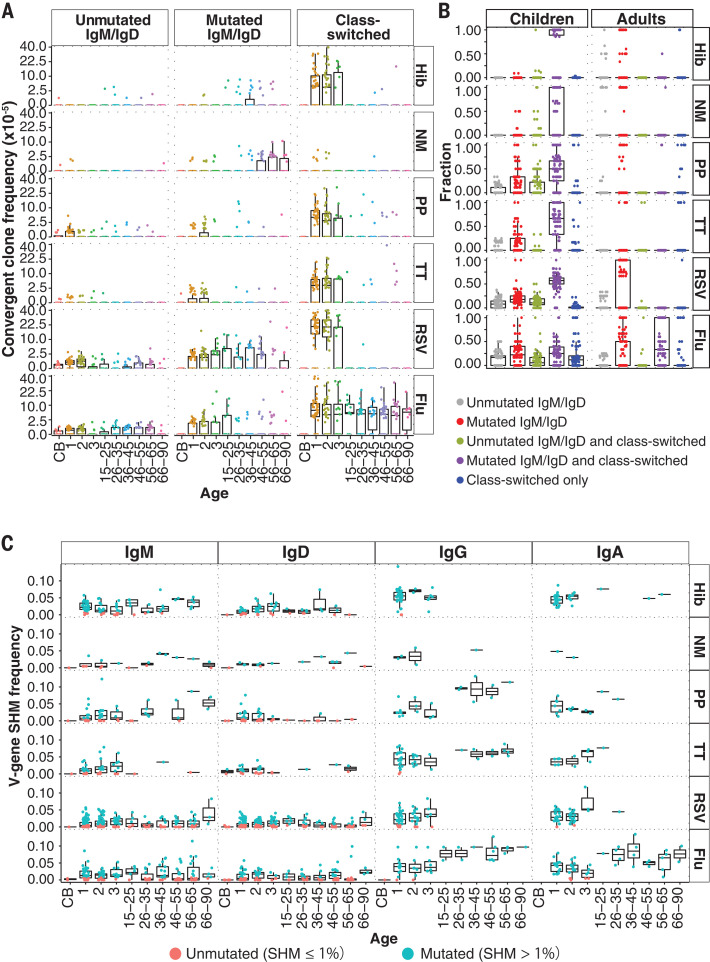
Frequency, class switching, and SHM of pathogen-specific convergent clones in children and adults. (**A**) Convergent clone frequencies for each pathogen, plotted on a square root scale. Ages given in years. CB, cord blood. (**B**) Fractions of convergent clones expressing unmutated IgM or IgD, mutated IgM or IgD, class-switched, or combinations of these. Children have significantly larger fractions of class-switched convergent clones with mutated IgM/IgD clone members (colored in purple) than do adults [*P* = 5.08 10^32^, 6.66 10^29^, 2.39 10^29^, 3.45 10^34^, and 1.71 10^41^ for Hib, NM, PP, TT, and RSV, respectively, by Wilcoxon-Mann-Whitney (WMW) test]. (**C**) Median IGHV gene SHM frequencies of each convergent clone in participants of different ages indicated in years. SHM frequencies of convergent clones expressing IgG or IgA were lower in children than in adults (*P* = 6.50 10^13^ and 1.96 10^8^, respectively; WMW test).

B cell clones fell into three groups: (i) nave clones containing only unmutated IgM or IgD (hereafter, unmutM/D); (ii) antigen-experienced IgM or IgD with median SHM over 1% and without class-switched members (hereafter, mutM/D); and (iii) antigen-experienced clones with class-switched members (hereafter, CS). As we hypothesized, CB samples showed the lowest convergent IGH frequencies, consistent with limited fetal pathogen or vaccine exposures (Fig. 1A). Convergent clones in children and adults were largely mutM/D or CS (Fig. 1A and fig. S1). In adult blood, convergent clones for Hib, NM, and RSV were predominantly mutM/D clones, whereas PP, TT, and flu clones were predominantly CS (fig. S2A). Adults over 45 years of age had elevated mutM/D B cell clone frequencies to NM, potentially from exposures preceding widespread NM vaccination (*20*). Unexpectedly, children had higher frequencies than adults of CS convergent clones for Hib, PP, TT, and RSV (fig. S2B), with mutated IgM or IgD also found in these clones (Fig. 1B). Convergent clone frequency in childrens blood was not significantly associated with vaccination timing (figs. S3 to S5 and table S3), indicating persistently elevated frequencies. Flu-specific convergent clone frequencies were comparable in children and adults (Fig. 1A), with age-related increases in IgG SHM potentially due to frequent exposures via vaccination or infection (Fig. 1C) (*18*).

**Fig. 2:**
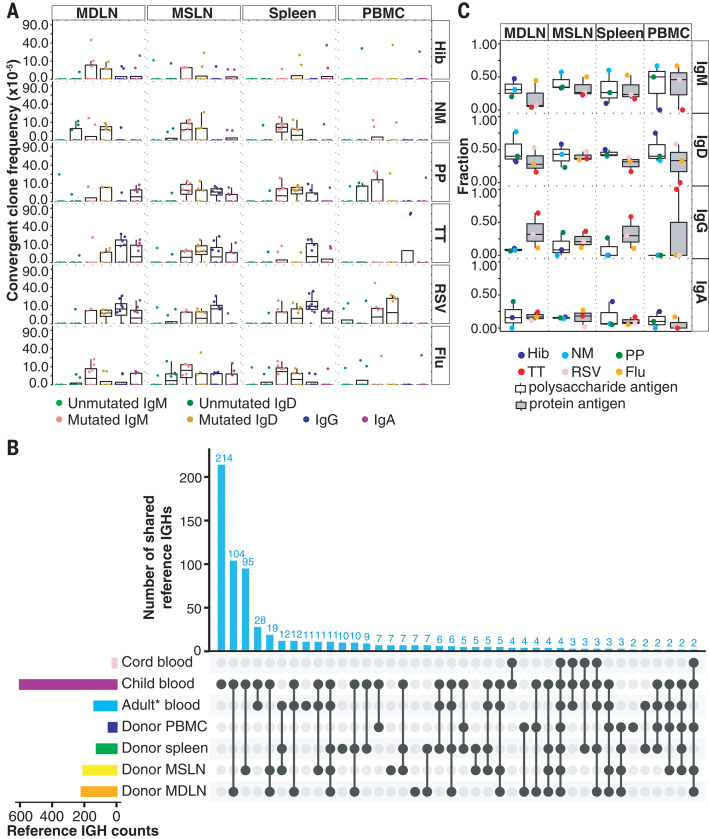
Convergent B cell clone distribution in tissues. (**A**) Convergent clone frequencies in adult blood (PBMC), MDLN, MSLN, and spleen. Frequencies are on a square root scale. Frequencies in tissues were higher than in blood (*P* = 0.00049, 0.0037, 0.016, 6.71 10^7^, 0.012, and 0.00017 for Hib, NM, PP, TT, RSV, and flu, respectively; WMW test). (**B**) Convergent antigen-specific IGH in CB and blood of children; healthy adults (Adult* blood); deceased organ donors (Donor PBMC); and donor spleen, MSLN, and MDLN. Vertical bars: reference antigenspecific IGH sequences per specimen combination. Left bars: total convergent IGH unique sequences per tissue. (**C**) Fraction of convergent clones containing the indicated isotypes in tissues. Some clones contain multiple isotypes. Compared with protein antigenspecific clones, polysaccharide-specific clones more frequently express IgM/D and less often express IgG (*P* = 0.035 and 0.0058, respectively; WMW test).

To test whether low frequencies of CS convergent clones in adult blood reflect preferential localization of clones in lymphoid tissues, we analyzed the blood, spleen, mediastinal lymph nodes (MDLN), and mesenteric lymph nodes (MSLN) of eight adult deceased organ donors. Lymph nodes and spleen showed greater clonal sharing with each other than with blood (fig. S6A), suggesting larger clone sizes in lymphoid tissues and limited recirculation. Each tissue was dominated by different clones (fig. S6B), and SHM correlated with the number of tissues a clone occupied (fig. S7), consistent with greater prior antigen exposure leading to wider tissue distribution (*21*). Convergent clone frequencies for Hib, NM, PP, TT, RSV, and flu were higher in adult lymph nodes and spleen than in blood (Fig. 2A). Adult lymph nodes and child blood shared more convergent clones than did adult and child blood, showing differing distributions of these clones in children and adults (Fig. 2B and fig. S8; *P* = 0.0001181, Fishers exact test). B cells specific for bacterial capsular polysaccharides are reported to be enriched in the spleen, and splenectomized patients are vulnerable to these bacteria (*16*, *17*). However, frequencies of convergent clones for Hib, NM, and PP are similar or higher in lymph nodes than in the spleen. Moreover, estimated B cell numbers are greater in human lymph nodes than the spleen (*22*, *23*), indicating that the spleen is not the sole reservoir of these clones. Convergent IGH for polysaccharides were usually IgM or IgD, with some CS clones for PP in lymph nodes and spleen (Fig. 2C). Thus, memory to these antigens spans a diversity of both lymphoid tissues and isotype expression.

**Fig. 3:**
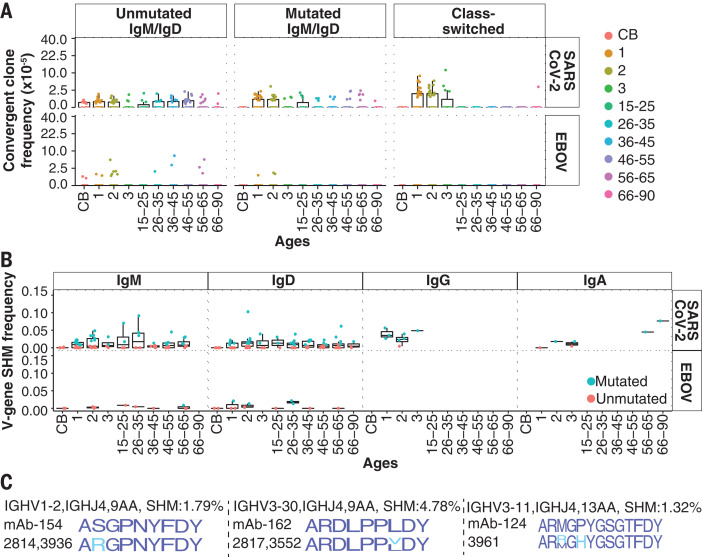
Convergent clones for SARS-CoV-2 and EBOV. (**A**) Convergent clone frequencies on a square root scale. CS and mutM/D convergent clone frequencies for SARS-CoV-2 are higher in children than in adults (*P* = 1.22 10^13^ and 0.0089, respectively; WMW test). (**B**) SHM frequencies of convergent clones for each isotype in participants of different ages (*x* axis). (**C**) CDR-H3 amino acid sequences of convergent IGH cross-reactive to SARS-CoV-2 and other HCoVs. Top row: CDR-H3 sequence logos for reported antigen-specific clones. Second row: sequence logos for convergent clones from children (blue indicates a match, cyan indicates sequence differences).

Recent reports describe SARS-CoV-2binding antibodies in prepandemic childrens blood (*12*, *24*). Such antibodies and other physiological distinctions are under investigation in adults and children (*25*) and could contribute to the generally milder COVID-19 disease in children. SARS-CoV-2 S-binding B cells in unexposed individuals have been analyzed in a former SARS-CoV patient (*26*), nave B cells of healthy individuals (*27*), and memory B cells in prepandemic donors (*26*, *28*). We detected rare convergent clones for EBOV, as unmutM/D in blood or tissues (Fig. 3A and fig. S9A). By contrast, convergent clones for SARS-CoV-2 (table S4) were more common in childrens blood. In 37 of 51 children, these clones displayed SHM with or without CS, indicating prior antigen experience (Fig. 3, A and B). Adult frequencies of SARS-CoV-2 convergent clones were lower in blood and lymphoid tissues compared with childrens blood, with few CS examples (Fig. 3A and fig. S9). Convergent clones specific for SARS-CoV-2 receptor binding domain (RBD) and other S domains showed similar distributions (fig. S10). Reference antibodies for SARS-CoV-2, EBOV, and the pathogens in Fig. 1 used a wide diversity of IGHV genes (fig. S11).

Three convergent clones from five children in this study, but none from adults, had IGH sequences highly similar to SARS-CoV-2 S-binding clones isolated from a prepandemic donor that were reported to weakly bind other human coronavirus (HCoV) spikes (*26*) (Fig. 3C). Three other clones from six children had IGHs identical to known SARS-CoV-2 binders (fig. S12). We expressed 19 monoclonal antibody (mAb) clones for SARS-CoV-2 (table S5) with IGH from participants in this study and reference light chains, and we identified 17 binders for SARS-CoV-2 S and S domains (Table 1). Four RBD binders showed >90% blocking of angiotensin-converting enzyme 2 (ACE2) binding to SARS-CoV-2 S (table S6). mAb FY11H1 showed evidence of S2 binding and did not block ACE2 binding. We characterized the breadth of mAb binding using a panel of HCoV spikes and SARS-CoV-2 viral variant RBDs and spikes. Three child-derived mAbs (FY7H1, FY7H2, and FY1H2) and one adult mAb (FY4H1) showed the strongest binding to B.1.1.7, B.1.351, and P.1 S and RBD variants (table S7). Cross-reactive binding to endemic HCoV spikes was very weak to absent for all mAbs, as previously noted for reference mAb-154 (similar to mAb FY13H1) isolated from a sorted cross-reactive B cell (*26*). The child-derived mAbs FY13H1 and FY9H2 had a higher, although still weak, signal for binding HKU1. Thus, childrens convergent coronavirus-binding B cells may have greater cross-reactivity than those of adults, in addition to having higher frequencies.

**Table 1:** Convergent mAb binding data for SARS-CoV-2 spike, RBD, and nucleocapsid (N) and endemic HCoV spikes. Testing by electrochemiluminescence immunoassay in duplicate wells, with the average arbitrary unit per milliliter (AU/ml) values displayed in the table. Antibodies with binding signal at least five standard deviations above the average of negative control antibodies (Neg1 to Neg5) are listed.

**mAb**	**CoV-2 S**	**CoV-2 RBD**	**CoV-2 S2**	**CoV-2 N**	**CoV S**	**HKU1 S**	**OC43 S**	**NL63 S**	**229E S**	**Source**
FY1H3	161.84	132.42	0.12	2.42	4.62	0.22	0.30	0.30	0.22	Children
FY3H1	158.05	130.83	0.08	0.89	1.00	0.10	0.17	0.17	0.11	Both
FY3H3	153.27	123.26	0.26	6.33	2.91	0.38	0.69	0.61	0.45	Children
FY3H2	150.57	127.35	0.24	0.87	1.18	0.10	0.16	0.14	0.09	Adults
FY7H1	149.78	125.82	0.12	1.41	4.21	0.10	0.19	0.19	0.13	Children
FY1H2	148.44	119.56	2.39	1.92	18.00	0.47	0.63	0.58	0.50	Children
FY13H1	147.29	119.22	0.15	3.55	0.39	1.41	0.47	0.29	0.25	Children
FY7H2	146.59	120.11	0.06	1.94	4.29	0.24	0.15	0.16	0.12	Children
FY4H1	131.23	116.83	0.19	2.27	1.32	0.25	0.39	0.27	0.23	Adults
FY8H1	114.20	107.56	0.03	0.80	2.08	0.12	0.09	0.07	0.04	Adults
FY9H1	91.02	94.74	1.39	3.62	5.87	0.75	0.56	0.31	0.30	Children
FY11H1	79.65	41.41	13.59	0.78	44.62	0.13	0.09	0.06	0.12	Both
FY6H1	79.09	71.33	0.54	5.22	2.88	0.45	0.27	0.21	0.24	Both
FY14H1	78.73	63.10	3.91	2.43	9.56	0.30	0.45	0.35	0.29	Adults
FY5H1	69.60	45.71	0.33	3.27	1.71	0.49	0.50	0.25	0.23	Children
FY9H2	53.53	13.86	3.83	2.42	10.03	1.46	0.35	0.23	0.25	Children
FY10H1	9.96	8.69	0.02	0.18	0.27	0.02	0.08	0.02	0.02	Adults
Neg5	0.52	0.01	0.00	0.00	0.02	0.00	0.00	0.00	0.00	Controls
Neg4	1.63	0.02	0.00	0.90	0.09	0.01	0.00	0.00	0.00	Controls
Neg3	1.22	0.02	0.00	0.03	0.07	0.00	0.00	0.00	0.00	Controls
Neg2	1.37	0.02	0.00	0.02	0.08	0.01	0.00	0.00	0.00	Controls
Neg1	0.87	0.02	0.00	0.01	0.04	0.00	0.00	0.00	0.00	Controls

Childhood immune responses are particularly important in an individuals life, as they form the initial memory B cell pool that shapes future responses (*29*). We find that in comparison to adults, children have higher frequencies of convergent B cell clones in their blood for pathogens they have encountered. Notably, prepandemic children also had class-switched convergent clones to SARS-CoV-2 and its viral variants, but not EBOV, at higher frequencies than adults. We hypothesize that previous HCoV exposures may stimulate cross-reactive memory, and that such clonal responses may have their highest frequencies in childhood. The caveats of our analysis are that convergent clones may not fully represent the properties of all pathogen-specific clones in an individual and that binding affinities for cross-reactivity that would be relevant in vivo are not known. Further study of the role of cross-reactive memory B cell populations in primary immune responses to related but divergent viruses as well as better understanding of the determinants of long-lived B cell memory and plasma cell formation will be important for ongoing improvement of vaccines to SARS-CoV-2, its viral variants, and other pathogens.

## Supplementary Materials

science.sciencemag.org/content/372/6543/738/suppl/DC1 Materials and Methods Figs. S1 to S12 Tables S1 to S9 References (*30*,*84*) MDAR Reproducibility Checklist

## Appendix

View/request a protocol for this paper from *Bio-protocol*.
